# In Vitro Biological Properties of Thiazole-Containing Cobalt(II) and Copper(II) Phthalocyanines: DNA Cleavage, Antioxidant, Antimicrobial, Antibiofilm, and Surface Anti-*Escherichia coli* Activities

**DOI:** 10.3390/ph19091459

**Published:** 2026-09-14

**Authors:** Nazlı Farajzadeh Öztürk

**Affiliations:** Department of Analytical Chemistry, Faculty of Pharmacy, Acibadem Mehmet Ali Aydinlar University, Istanbul 34752, Türkiye; nazli.ozturk@acibadem.edu.tr

**Keywords:** phthalocyanine, thiazole, antioxidant, antimicrobial, DNA cleavage, wound

## Abstract

**Background/Objectives**: Given the excellent biochemical and optical properties of phthalocyanines, this study aims to develop new phthalocyanine-based bioagents for multidisciplinary applications. **Methods**: 3-((4-(4-fluorophenyl)thiazol-2-yl)thio)phthalonitrile was synthesized by coupling 3-nitrophthalonitrile with 4-(4-fluorophenyl)thiazole-2-thiol, then cyclotetramerized in the presence of cobalt(II) or copper(II) chloride to afford phthalocyanines (TCoPc, TCuPc). Unsubstituted cobalt(II) and copper(II) phthalocyanines (CoPc, CuPc) were prepared as described in the literature and used as reference compounds. The DNA-cleavage, antioxidant, antimicrobial, antibiofilm, and surface antimicrobial activities of the new phthalocyanines were evaluated at different concentrations. The biological activities of CoPc and CuPc were investigated for the first time to examine the effects of metal ions and substituents on the phthalocyanine ring. **Results**: All phthalocyanines caused complete DNA degradation at 125–250 mg/L. Their antioxidant activity ranged from 16.44% to 69.54% at 6.25 mg/L and increased to 28.70–77.91% at 100 mg/L (TCuPc > TCoPc > CoPc > CuPc). TCuPc exhibited the strongest antimicrobial activity, with MIC values of 16 mg/L against *S. aureus*, *L. pneumophila*, and *B. cereus*, and 32 mg/L against *E. hirae*, *P. aeruginosa*, and *E. coli*. TCuPc also achieved the highest biofilm inhibition, up to 92.35% against *S. aureus* and 87.24% against *P. aeruginosa* at 200 mg/L, and the greatest surface antimicrobial inhibition, reaching 86.78% in the dark and 95.71% under photodynamic conditions at 50 mg/L. **Conclusions**: Substitution with thiazole improved the biological activities of metal phthalocyanines, and copper(II) coordination provided an additional benefit over cobalt(II). TCuPc emerges as the most promising candidate, justifying further clinical research.

## 1. Introduction

DNA cleavage, a key process in biology and genetic engineering, is primarily mediated by enzymes. For example, topoisomerases cut DNA strands to resolve issues during replication and transcription, while restriction enzymes protect against viruses by cleaving foreign DNA or degrading cellular DNA during apoptosis [1,2]. Developing highly specific artificial nucleases that cut DNA is crucial for advancing DNA-manipulation tools for biotech and pharma. Recently, phthalocyanine derivatives have attracted attention as potential multidisciplinary bioagents owing to their strong interactions with DNA. Arslantaş and Ağırtaş synthesized new diethyl (phenyl)malonate-containing metal {M = Zn(II) and Co(II)} phthalocyanines and studied their DNA interaction activities. The resultant complexes significantly fragmented DNA molecules [3]. Yılmaz et al. prepared metal phthalocyanine (MPc)-modified poly(acrylonitrile-co-vinyl acetate) (PAN) nanofibers (MgPc/PAN and ZnPc/PAN) and examined their DNA cleavage activities. In contrast to unmodified PAN nanofibers, MPc/PAN nanofibers completely cleaved DNA molecules at the tested concentrations [4].

Metabolic, cardiovascular, and neurodegenerative diseases originate from oxidative stress, which damages biomolecules such as lipids, proteins, and DNA through excessive ROS production. Antioxidants, including plant phytochemicals, vitamins, and marine compounds, neutralize oxidative stress caused by prooxidants such as ROS. Generally, they help maintain the body’s redox balance by reducing oxidative stress. The preventive role of phthalocyanines in oxidative stress-related diseases has been studied over the last few decades. Samsunlu et al. prepared new tetra-substituted metal phthalocyanines {M = Zn(II), Cu(II), and Co(II)} containing tetraethyleneglycol monomethyl ether chains at peripheral and non-peripheral positions and studied their antioxidant activities. The cobalt(II) phthalocyanines, especially the non-peripheral one, exhibited the highest antioxidant activities [5].

Antimicrobials are natural or synthetic compounds that inhibit or kill bacteria, fungi, viruses, and some parasites. They are a major focus in pharmaceutics, microbiology, and biotechnology. Antimicrobial surfaces, designed to prevent contamination, are crucial in hospitals, biomedical devices, dental materials, and the food industry. However, antimicrobial resistance, driven by the misuse or overuse of antibiotics and disinfectants, has reduced their effectiveness [6,7]. Biofilm formation can be harmful, especially in medical and industrial settings. Biofilms provide a protective environment for bacteria and enable the formation of complex communities. Research on biofilm formation and removal is vital for developing new therapies against resistant pathogens [8]. Among the diverse antimicrobial candidates, heterocyclic structures, especially phthalocyanines, have attracted researchers’ interest in recent years. Türkan et al. synthesized two new tetra-substituted cobalt(II) and zinc(II) phthalocyanines bearing (ethylenediamine-N,N′,N′-triacetic acid-N-2-ethyl)oxy (ETAEO) groups at the peripheral positions and investigated their antibacterial activities against *E. coli* and *S. aureus*. Both phthalocyanines were more effective against the Gram(−) bacterium *E. coli* than against the Gram(+) bacterium *S. aureus* [9].

Plant-derived phytochemicals, microbial metabolites, antimicrobial peptides, and synthetic compounds are extensively studied as alternative antimicrobial agents [6,10]. Photodynamic antimicrobial therapy effectively kills microorganisms and limits the emergence of resistance. In this approach, a photosensitizer is excited by light, generating reactive oxygen species (ROS) that damage microbial components and lead to cell death [11]. Metal phthalocyanines are among the most effective photosensitizers, owing to their π-conjugated systems, which confer strong absorption in the visible and near-infrared regions. They efficiently produce singlet oxygen, making them popular in photodynamic antimicrobial therapy and surface coatings [12]. Kılıçarslan et al. prepared two new peripherally hexyl benzoate-tetrasubstituted copper(II) and nickel(II) phthalocyanines (4HEBCu and 4HEBNi). The antimicrobial activities of these phthalocyanines were examined against *S. aureus* and *P. aeruginosa*, with and without irradiation. They exhibited greater inhibitory activity after light exposure [13].

Phthalocyanines (Pcs) feature a two-dimensional 18π-electron ring, resulting in high electron-transfer rates [14,15]. They exhibit remarkable thermal resistance, chemical stability, and optical properties, making them fascinating materials for many scientific and technological fields. In particular, the ability to alter the structure by introducing diverse substituents into the ring and/or inserting metal ions at the ring center is the most important characteristic of phthalocyanines, as this flexibility is beneficial for designing materials with specific features [16,17,18,19,20,21,22,23].

Phthalocyanines (Pcs) are formed from four isoindole units. An indole unit consists of a six-membered benzene ring fused to a five-membered pyrrole ring [24] and is found in many pharmacologically important molecules, such as neurotransmitters [25], amino acids [26], and hormones [27]. Indole-based compounds often exhibit outstanding biological properties [28,29]. In addition to indoles, thiazoles are fundamental heterocyclic molecules found in many potent antitubercular, antioxidant, anti-inflammatory, antitumor, anti-HIV, anticonvulsant, antibacterial, and antifungal agents [30,31,32,33,34,35,36,37,38,39,40,41,42]. For example, Vijesh et al. reported the synthesis and antimicrobial activities of several 2,4-disubstituted thiazole Schiff bases containing a pyrazole moiety. All the compounds exhibited antibacterial activity against *E. coli*, *B. subtilis*, *S. aureus*, and *P. aeruginosa* [43]. Furthermore, Mayhoub et al. tested the antiviral activity of a new-generation methyl 4-(dibromomethyl)-2-(4-chlorophenyl)thiazole-5-carboxylate against yellow fever virus using a cell-based assay [44]. Likewise, Yadla et al. investigated the antioxidant activity of 2,4-dioxo-1,3-thiazolidine thiazoles using the DPPH method. The highest antioxidant activity was observed for the para-bromo derivative, comparable to that of luteolin and ascorbic acid [45]. As a result, thiazoles can also be used to design promising therapeutic agents with higher efficacy, better pharmacokinetics, and fewer side effects.

Regarding the individual biological activities of phthalocyanine and thiazole derivatives, this study primarily aims to assess whether introducing thiazole groups into the phthalocyanine ring enhances its biological activity. Additionally, cobalt and copper were chosen as the central metal cations because most cobalt(II) and copper(II) phthalocyanines have demonstrated good biological activities [46,47]. Therefore, a new phthalonitrile and its metal phthalocyanine derivatives, {Co(II) and Cu(II)}, containing thiazole groups were synthesized in this study. Additionally, unsubstituted Co(II) and Cu(II) phthalocyanines were prepared as previously reported [10,11,12]. The effects of metal ions and substituents on the DNA-cleavage, antioxidant, and antimicrobial activities of the phthalocyanine ring were extensively evaluated by examining the biological activities of all the prepared substituted and unsubstituted phthalocyanines. The results obtained were also compared with the literature.

## 2. Results and Discussion

### 2.1. Synthesis and Characterization

**Scheme 1:**
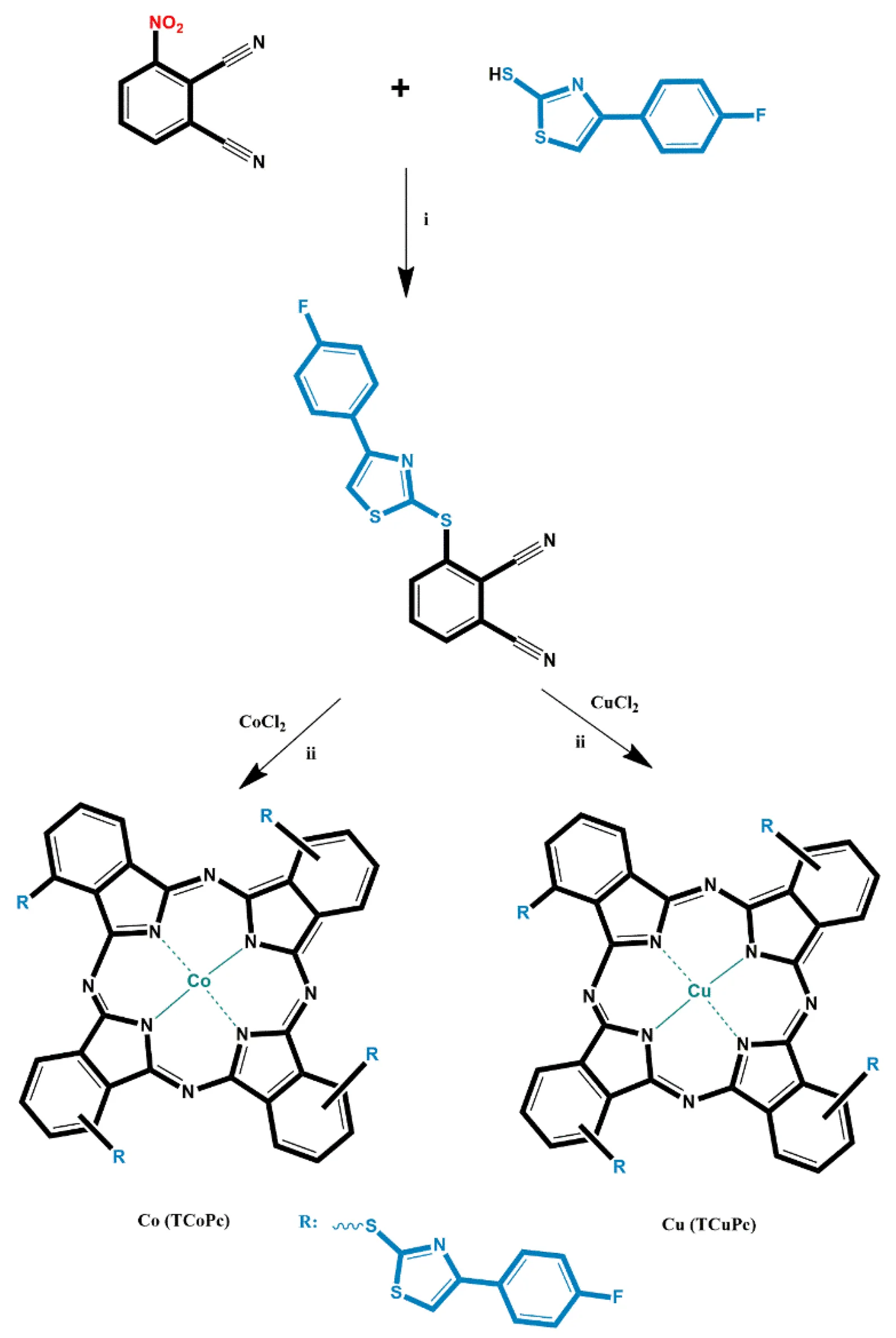
The synthetic route for the phthalonitrile derivative and compounds (TCoPc and TCuPc): (i) K_2_CO_3_, N,N-dimethyl formamide, 70 °C, 72 h; (ii) N,N-dimethyl aminoethanol, reflux temperature, 24 h.

The synthetic route to the phthalonitrile derivative and the compounds (TCoPc and TCuPc) is shown in Scheme 1. 3-Nitrophthalonitrile and 4-(4-fluorophenyl)thiazole-2-thiol were coupled via an aromatic substitution reaction to afford 3-((4-(4-fluorophenyl)thiazol-2-yl)thio)phthalonitrile. The pure product was recrystallized from ethanol and characterized using elemental analysis (Thermo Finnigan Flash EA 1112, Milan, Italy), FT-IR spectroscopy (Perkin-Elmer Spectrum One spectrometer, Shelton, Connecticut, USA), and ^1^H NMR spectroscopy (Agilent VNMRS 500 MHz spectrometer, Palo Alto, Santa Clara, CA, USA). In the FT-IR spectrum (Figure S1a), the aromatic C–H and nitrile bands appeared at 3043 and 2227 cm^−1^, whereas the C-F and C-S-C bands were observed at 1251 and 1046 cm^−1^, respectively [48]. In the ^1^H NMR spectrum (Figure S2), the singlet at 8.27 ppm was assigned to the single proton on the thiazole ring, whereas the signals of the two protons on the phthalonitrile ring near the nitrile groups appeared as doublets at around 8.22–8.19 and 8.10–8.07 ppm, respectively. The signals of the remaining protons overlapped and appeared as a multiplet between 7.98 and 7.92 ppm, corresponding to 5 protons [48]. All the data were consistent with the proposed structure.

**Figure 1:**
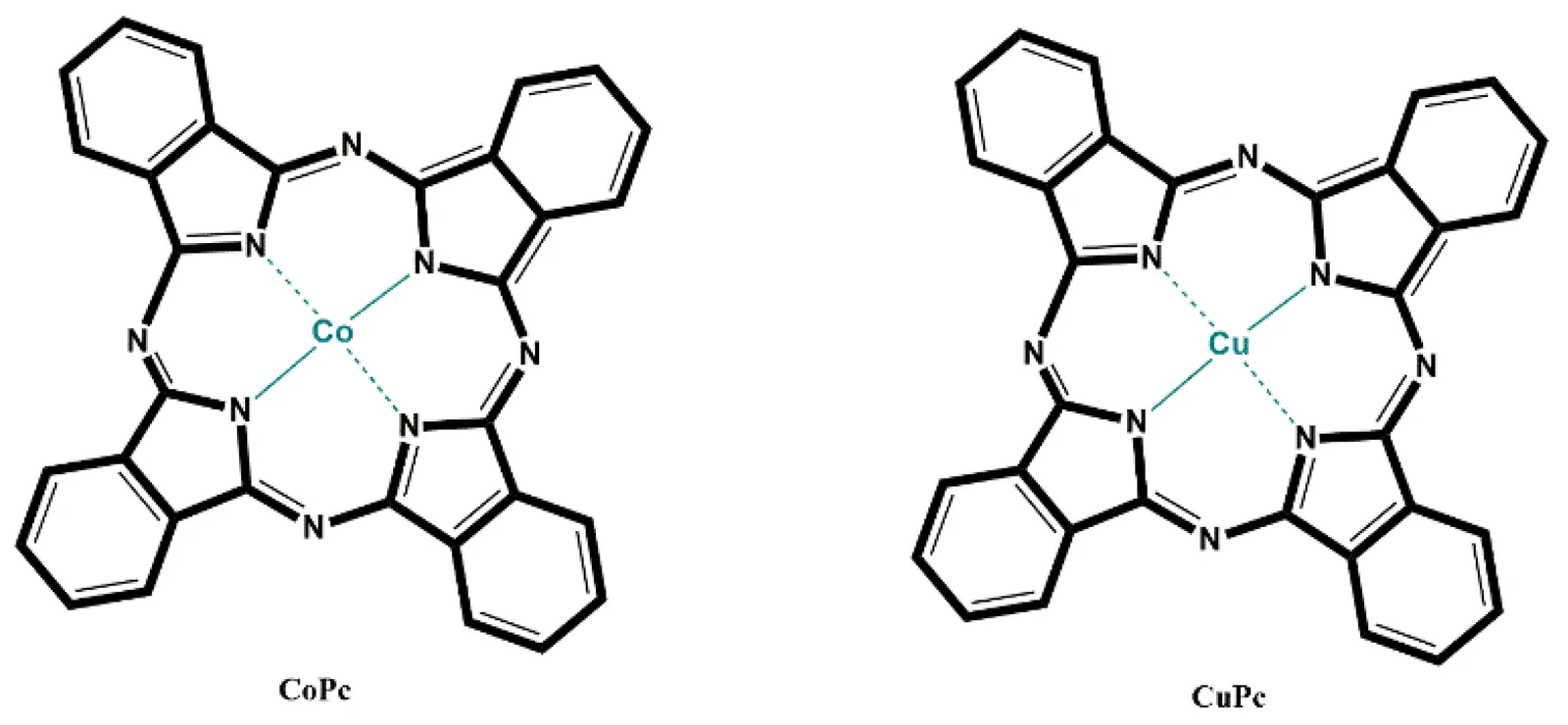
The chemical structures of compounds (CoPc and CuPc).

The resulting phthalonitrile derivative was cyclotetramerized in the presence of the corresponding metal chloride via a one-step templating mechanism to afford metal phthalocyanines (TCoPc and TCuPc). The resulting compounds were purified by column chromatography on silica gel (stationary phase) eluted with a mixture of *n*-hexane/tetrahydrofuran (3:1 *v*/*v*, (mobile phase). We characterized the obtained phthalocyanines by FT-IR, UV-vis (Scinco Lab Pro Plus ultraviolet-visible diode-array spectrophotometer, Seoul, South Korea), and MALDI-TOF (Perkin-Elmer Clarus 500 gas chromatography-mass spectrometry, Shelton, Connecticut, United States) spectroscopy. In the FT-IR spectra of the phthalocyanines (Figure S1b,c), the aromatic C–H, C-F, and C-S-C bands were observed at approximately 3035, 1250, and 1073 cm^−1^, respectively [48]. The disappearance of the nitrile bands at approximately 2227 cm^−1^ confirmed the successful cyclotetramerization of the phthalonitrile molecules. In the UV-vis spectra of compounds (TCoPc and TCuPc) (Figures S3 and S4), the Q-bands corresponding to π-π* transitions from HOMO (highest occupied molecular orbital) to LUMO (lowest unoccupied molecular orbital) were observed at 681 and 684 nm, respectively. The B-bands, assigned to π-π* transitions from deeper π-orbitals to LUMO, appeared at 357 and 351 nm, respectively [14,15,16,17,18,19,20,21,22,23,24]. These results confirmed the successful synthesis of TCoPc and TCuPc. Generally, the mass spectrum of a compound should be assigned to its ionized intact structure; however, differences may arise from the medium (H_2_O or moisture), basicity (e.g., potassium hydroxide) or acidity (e.g., acetic acid), and the matrix in which the measurement is performed. Some of these differences have been reported in the literature [49,50]. In this study, the MALDI-TOF analysis was performed twice to ensure reproducibility. Although the molecular ion peak of TCoPc is targeted at *m*/*z* 1408.51, an ion peak at *m*/*z* 1327.90 was detected in the MALDI-TOF analysis and attributed to the parent compound. This ion may result from loss of a substituent, with retention of two potassium ions and three hydroxide ions. This assignment is supported by the calculated molecular ion weight of [M − C_9_H_5_FNS_2_ + 2K + 3OH]^+^, which is *m*/*z* 1327.90 and consistent with the ion observed in the MALDI-TOF spectrum (Figure S5). The molecular ion peak of TCuPc is targeted at *m*/*z* 1413.12; an ion peak at *m*/*z* 1518.61 was observed in the MALDI-TOF analysis and assigned to the parent compound. This ion peak may arise from retention of three sodium ions and two water molecules. This assignment is supported by the calculated molecular ion weight of [M + 3Na + 2H_2_O]^+^, which is *m*/*z* 1518.61 and consistent with the ion appearing in the MALDI-TOF spectrum (Figure S6). Moreover, unsubstituted cobalt(II) (CoPc) and copper(II) (CuPc) phthalocyanines were prepared as described in the literature [10,11,12]. The chemical structures of CoPc and CuPc are shown in Figure 1.

### 2.2. Biological Studies

**Figure 2:**
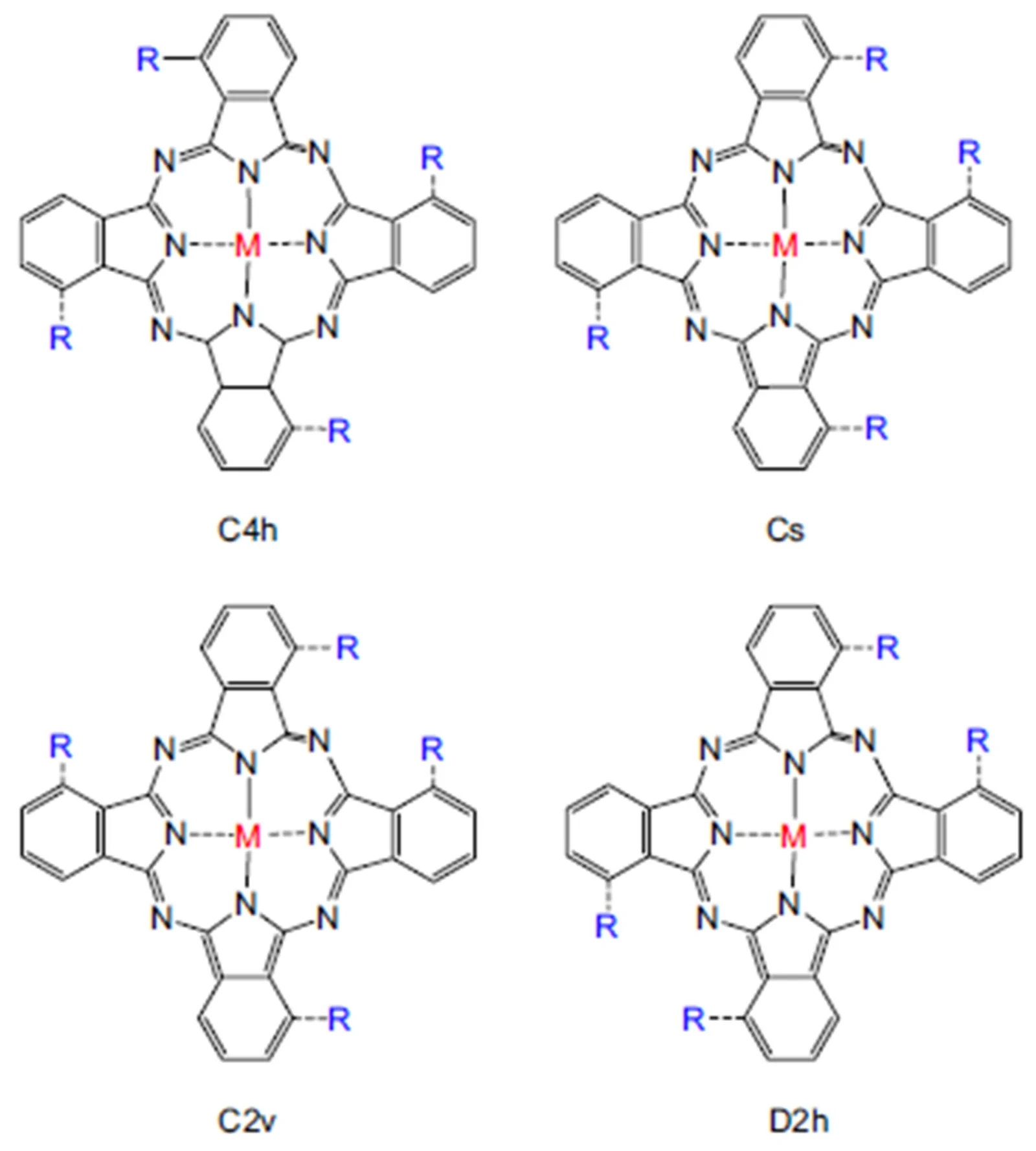
The isomeric mixture of compounds (TCoPc (M = Co) and TCuPc (M = Cu)); R: 4-(4-fluorophenyl)thiazol-2-yl)thio substituent.

Phthalocyanines synthesized via the cyclotetramerization of mono-substituted phthalonitriles typically form an isomeric mixture (symmetry; respective relative yields: D2h, C4h, C2v, and Cs) (Figure 2). This mixture tends to be more soluble than those derived from di-substituted phthalonitriles with identical substituents, owing to a higher dipole moment and reduced aggregation. A few studies have reported the successful use of chromatographic techniques to separate trace amounts of these structural isomers with D2h, C4h, C2v, and Cs symmetry in ratios of 1:1:2:4, respectively [14,15]. No further separation was performed on the resulting mixture, and the biological studies were carried out on the mixture exhibiting higher solubility and lower aggregation.

#### 2.2.1. DNA Cleavage Activity

**Figure 3:**
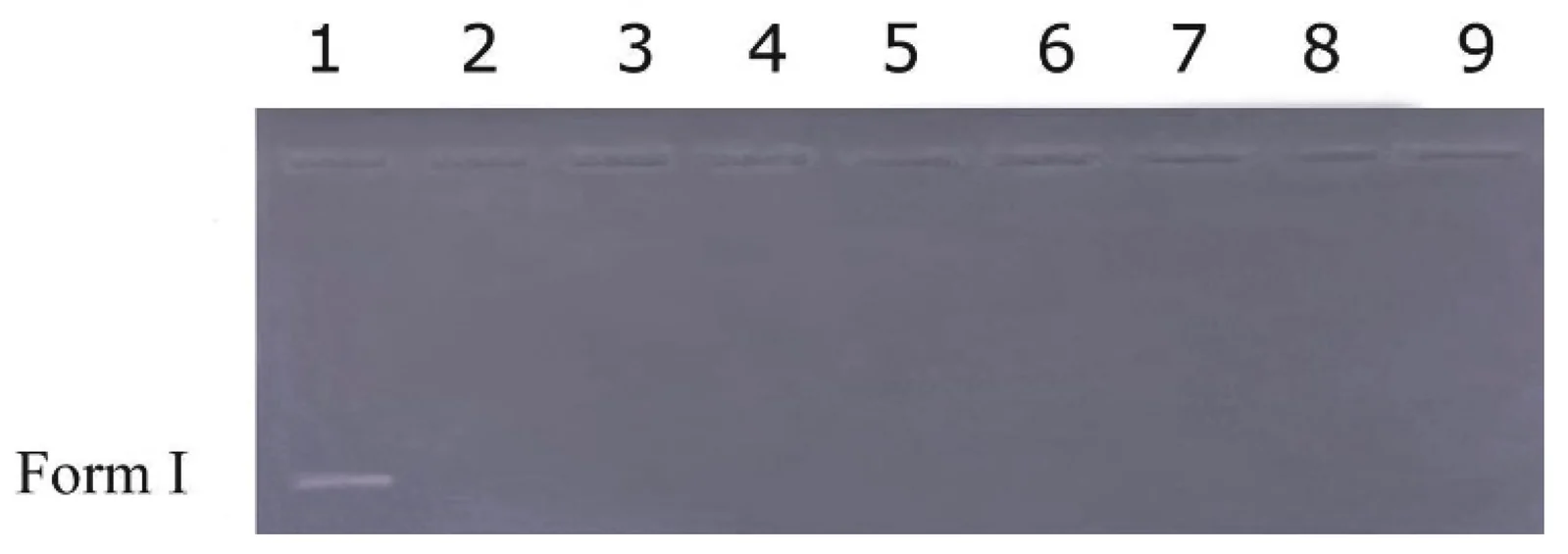
DNA cleavage activities of compounds (CoPc, TCoPc, CuPc, and TCuPc). Lane 1, pBR 322 DNA + DMSO; Lane 2, pBR 322 DNA + 125 mg/L of CoPc; Lane 3, pBR 322 DNA + 250 mg/L of CoPc; Lane 4, pBR 322 DNA + 125 mg/L of TCoPc; Lane 5, pBR 322 DNA + 250 mg/L of TCoPc; Lane 6, pBR 322 DNA + 125 mg/L of CuPc; Lane 7, pBR 322 DNA + 250 mg/L of CuPc; Lane 8, pBR 322 DNA + 125 mg/L of TCuPc; Lane 9, pBR 322 DNA + 250 mg/L of TCuPc.

The DNA-cleavage activity of MPcs is typically influenced by the redox properties of their central metal ions. The extended π-conjugated phthalocyanine macrocycle promotes noncovalent interactions with DNA, including π–π stacking, electrostatic attractions, and groove binding, which depend on the peripheral substituents and the complex’s charge. These interactions position MPc molecules near the DNA backbone and bases, thereby enabling localized chemical reactions with the nucleic acids [51,52,53,54]. In this study, the DNA-cleavage activities of compounds (CoPc, TCoPc, CuPc, and TCuPc) were investigated, and the results are shown in Figure 3. The band in lane 1 is clearly visible and serves as the control, containing only DNA molecules and DMSO. In lanes (2–9), DNA bands are not visible because the test compounds completely cleaved the DNA molecules. As the DNA molecules were converted into oligonucleotides and/or mononucleotides, no bands were formed, and slight blurring was present. All the compounds caused complete DNA degradation at 125 mg/L and 250 mg/L. These results indicate that the test compounds exhibited highly effective DNA-cleavage activities. Özel et al. (2016) reported the preparation and characterization of new water-soluble, peripherally tetra-substituted phthalocyanines and tested their DNA-cleavage ability [55]. They found that the three titanium(IV) phthalocyanines bound to and cleaved DNA molecules. Çuhadar et al. (2023) studied the DNA-cleavage ability of non- peripherally and peripherally substituted copper(II) and manganese(III) phthalocyanines [56]. Non-peripherally and peripherally substituted copper(II) phthalocyanine exhibited complete DNA fragmentation at 200 mg/L, and peripherally substituted manganese(III) phthalocyanine caused complete DNA fragmentation at 100 and 200 mg/L. Previous studies have demonstrated that metal phthalocyanines can interact strongly with DNA through external binding, stacking, and electrostatic interactions, supporting the possibility that DNA–MPc association contributes to the observed cleavage activity [19,57]. Amitha and Vasudevan (2020) tested the DNA-binding and cleavage activities of CuBBPc, ZnBBPc, and their quaternized derivatives (qCuBBPc and qZnBBPc) [58]. The compounds possessed both DNA-binding and DNA-cutting abilities. The DNA-cleavage results obtained in this study revealed that compounds (CoPc, TCoPc, CuPc, and TCuPc) can be evaluated as DNA-binding agents, providing a strong basis for further in vitro/in vivo studies.

#### 2.2.2. Antioxidant Activity

**Figure 4:**
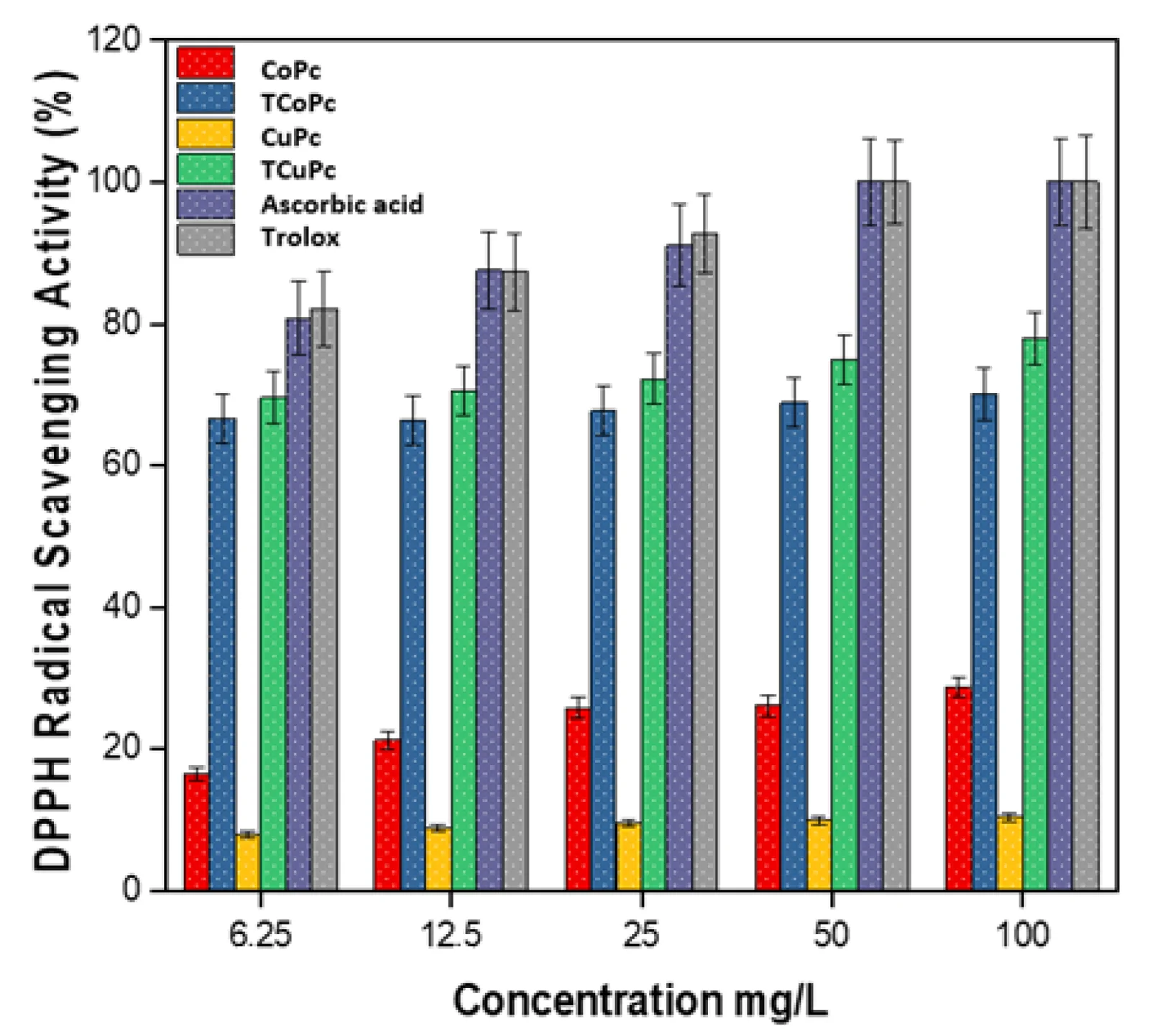
Antioxidant activities of compounds (CoPc, TCoPc, CuPc, and TCuPc).

In this study, the DPPH radical-scavenging activity of compounds (CoPc, TCoPc, CuPc, and TCuPc) was investigated. The results are shown in Figure 4 and listed in Table 1. All tested compounds exhibited antioxidant activity that did not change significantly with increasing concentration over the tested range (6.25–100 mg/L), indicating slight dose dependence. The antioxidant activities were 16.44% ± 0.89 for CoPc, 66.66% ± 3.49 for TCoPc, 8.00% ± 0.45 for CuPc, and 69. 54% ± 3.67 for TCuPc at the lowest concentration (6. 25 mg/L). The antioxidant activities of compounds (CoPc, TCoPc, CuPc, and TCuPc) were 28.70% ± 1.48, 70.07% ± 3.76, 10.37% ± 0.58, and 77.91% ± 3.68 at 100 mg/L, respectively. Accordingly, the antioxidant activity was ranked TCuPc > TCoPc > CoPc > CuPc throughout the concentration range. Recently, the antioxidant activities of metal phthalocyanine complexes have been extensively investigated. Their radical-scavenging properties vary significantly with both the metal center and the peripheral substituents. Günsel et al. (2019) synthesized water-soluble phthalocyanine derivatives and tested their DPPH radical-scavenging activities [59]. The highest antioxidant activity was 28. 3% for the cobalt(II) phthalocyanine containing 1(4),8(11),15(18),22(25)-tetrakis(8-(2,3-dicyanophenoxy) quinoline-5-sulfonic acid) at 500 mg/L. Similarly, 2-mercaptobenzimidazole-substituted phthalocyanine complexes exhibited effective radical-scavenging activities [60]. Baran et al. (2020) reported that chalcone-substituted zinc(II) and cobalt(II) phthalocyanines displayed 39.65% and 34.99% radical-scavenging activities at 100 mg/L, respectively [61]. Regarding the underlying mechanism, the radical-scavenging activity of metallophthalocyanines can be attributed to their extended π-conjugated macrocyclic structure and the electronic properties of the central metal ion. Depending on the chemical structure of the complex and the radical species involved, radical neutralization may occur through electron-transfer and/or hydrogen-atom-transfer processes. The central metal ion can modulate the electron density, redox properties, and electron-transfer capability of the phthalocyanine macrocycle, thereby influencing its overall radical-scavenging efficiency. Differences among the studied phthalocyanines may therefore be related to their distinct electronic structures, redox properties, molecular aggregation behavior, and accessibility of reactive sites. The significantly higher antioxidant activities of compounds TCoPc and TCuPc confirmed that their structural properties enhance their radical-scavenging capacity. Structural factors, such as electron- or hydrogen-donor capacity or degree of conjugation, may play a role in the antioxidant mechanism of compounds TCoPc and TCuPc against the DPPH radical.

#### 2.2.3. Antimicrobial Activity

Disk diffusion and broth microdilution are standard in vitro methods to evaluate antimicrobial activity. These methods enable qualitative and quantitative assessment of a compound’s effectiveness against microorganisms. The antimicrobial activities of compounds (CoPc, TCoPc, CuPc, and TCuPc) were investigated by determining minimum inhibitory concentrations (MICs) against various Gram-negative, Gram-positive, and yeast species. The results are summarized in Table 2. Accordingly, compound TCuPc exhibited the highest antimicrobial activity against the tested microorganisms. Compound TCuPc had a strong inhibitory effect, with a relatively low MIC of 16 mg/L, particularly against *B. cereus*, *L. pneumophila*, and *S. aureus*. Additionally, the MIC values of compound TCuPc were calculated to be 32 mg/L against *E. coli*, *P. aeruginosa*, and *E. hirae*. The antibacterial activities of compounds CoPc and TCoPc were generally moderate (MIC values: 64–128 mg/L) against most bacterial species; however, those of compound CuPc were higher against *P. aeruginosa*, *E. hirae*, and *S. aureus*. All the phthalocyanines exhibited similar antifungal activity against *Candida albicans* (64 mg/L) and *C. tropicalis* (64–128 mg/L). The reference antimicrobial agents had substantially lower MICs than the tested phthalocyanine derivatives. Fluconazole (FCZ) had an MIC of 1 mg/L against both *Candida albicans* and *Candida tropicalis*. These values align with the generally low MIC range reported for fluconazole against susceptible *Candida* isolates using standardized broth microdilution methods. Ampicillin (AMP) had an MIC of 0.5 mg/L against *E. coli*, *P. aeruginosa*, *L. pneumophila*, *E. hirae*, and *S. aureus*, whereas the MIC increased slightly to 1 mg/L against *B. cereus*. The low MICs for AMP confirmed its strong antibacterial activity and provided an appropriate reference for comparison with the phthalocyanine-based compounds. Türkan et al. (2021) prepared two new cobalt(II) and zinc(II) phthalocyanines containing ((ethylenediamine-N,N′,N′-triacetic acid-N-2-ethyl)oxy) (ETAEO) groups [9]. They examined their antibacterial activity against *E. coli* and *S. aureus*. The zinc(II) phthalocyanine derivative exhibited higher antibacterial activity than the cobalt(II) phthalocyanine. The antimicrobial activity of metallophthalocyanines may involve several complementary mechanisms rather than a single pathway. First, the extended π-conjugated structure and the physicochemical properties of the peripheral substituents can facilitate interactions with the bacterial cell envelope. Depending on molecular charge and structure, metallophthalocyanines may interact with negatively charged components of bacterial surfaces, including membrane phospholipids and other anionic cell-envelope constituents. These interactions can alter membrane organization and permeability, leading to leakage of intracellular components and disruption of essential cellular functions. Abalintsina et al. synthesized new cobalt(III), nickel(III), copper(II), and zinc(II) phthalocyanines bearing pyridine and thiocyanate groups [62]. The antimicrobial activities of the phthalocyanines were evaluated against four bacterial strains (*E. coli*, *K. pneumonia*, *S. typhi*, and *S. flexneri*) and four fungal strains (*C. albicans*, *C. parapsilopsis*, *C. tropicalis*, and *C. neoformans*). Although the cobalt(III) phthalocyanine exhibited higher activity than the reference drugs (doxycycline and Penicillin), the copper(II) complex displayed higher activity than the reference antibiotics (doxycycline and nystatin). Although the studied phthalocyanines had lower antimicrobial activities than fluconazole and ampicillin, their antimicrobial activities (particularly that of TCuPc) were comparable to those of other phthalocyanines reported in the literature. This supports their potential as next-generation antimicrobial agents following further research.

#### 2.2.4. Biofilm İnhibitory Activity

**Figure 5:**
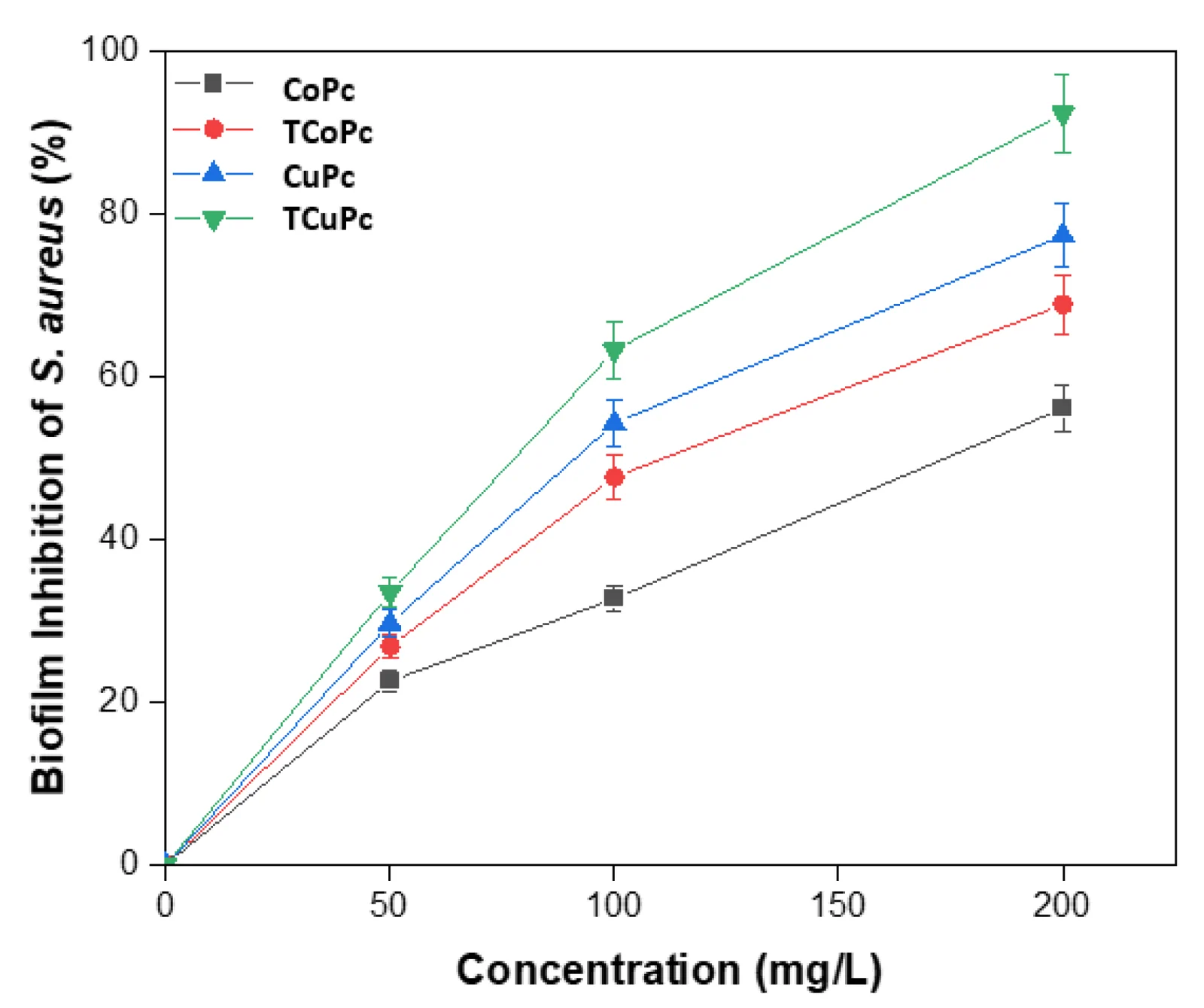
Biofilm inhibition of compounds (CoPc, TCoPc, CuPc, and TCuPc) against *S. aureus*.

**Figure 6:**
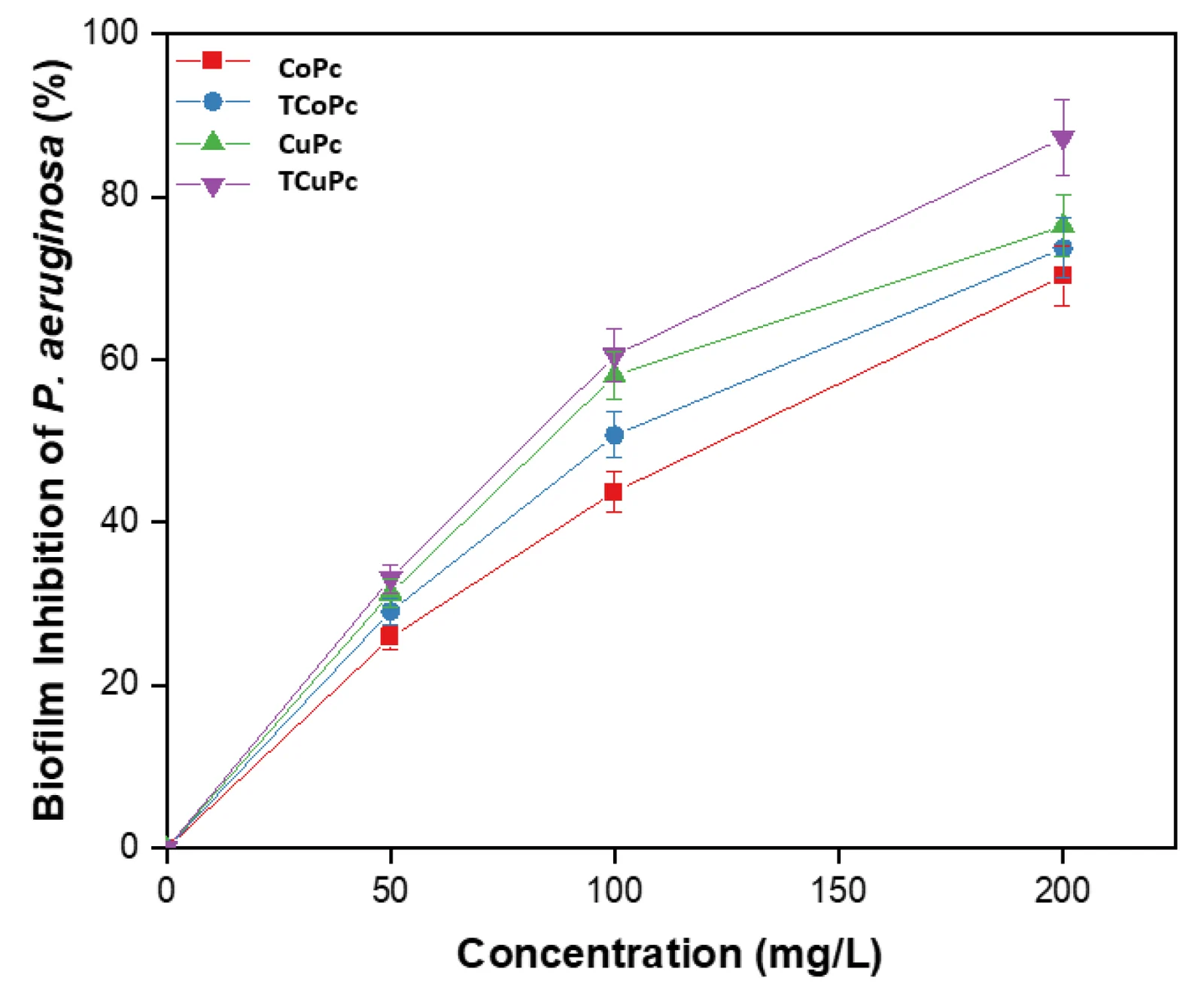
Biofilm inhibition of compounds (CoPc, TCoPc, CuPc, and TCuPc) against *P. aeruginosa*.

In this study, the inhibitory activities of compounds (CoPc, TCoPc, CuPc, and TCuPc) against biofilm formation by *S. aureus* and *P. aeruginosa* were evaluated at different concentrations (50, 100, and 200 mg/L). The results are depicted in Figure 5 and Figure 6, respectively. The data are also summarized in Table 3. The data indicated that all the metal phthalocyanines suppressed biofilm formation in a concentration-dependent manner. In general, a measurable degree of inhibition was observed even at 50 mg/L for the tested compounds against *S. aureus*. Compound CoPc exhibited 22.58% ± 1.27 inhibition, while compound TCoPc displayed 26.83% ± 1.51, CuPc 29.67% ± 1.67, and TCuPc 33.48% ± 1.74 inhibition at 50 mg/L. The biofilm inhibition activities were 32.71% ± 1.45 and 56.15% ± 2.84 for CoPc, 47.64% ± 2.63 and 68.91% ± 3.71 for TCoPc, 54.26% ± 2.79 and 77.42% ± 3.88 for CuPc, and 63.27% ± 3.41 and 92.35% ± 4.86 for TCuPc at 100 mg/L and 200 mg/L, respectively. Similarly, increasing the concentrations of compounds (CoPc, TCoPc, CuPc, and TCuPc) enhanced biofilm inhibition against the Gram-negative bacterium *P. aeruginosa*. The biofilm inhibition activities of compounds (CoPc, TCoPc, CuPc, and TCuPc) were 25.93% ± 1.39 and 43.79% ± 2.46 for CoPc, 29.11% ± 1.59 and 50.74% ± 2.79 for TCoPc, 31.37% ± 1.67 and 58.04% ± 2.96 for CuPc, and 33.15% ± 1.75 and 60.53% ± 3.21 for TCuPc at 50 mg/L and 100 mg/L, respectively. The highest biofilm inhibition, 87.24% ± 4.67, was observed with compound TCuPc against *P. aeruginosa* at 200 mg/L. The same concentration of CoPc, TCoPc, and CuPc exhibited biofilm inhibition rates of 70.28% ± 3.68, 73.68% ± 3.65, and 76.42% ± 3.72, respectively. Although Gram-negative *P. aeruginosa* is known for its resistance to antibacterial agents due to its outer membrane structure, phthalocyanines also exhibit strong biofilm inhibition against this microorganism. Çuhadar et al. (2023) tested the antibiofilm activity of some new zinc(II), copper(II), and manganese(III) phthalocyanines against *S. aureus* and *P. aeruginosa* [56]. Peripherally tetra-substituted zinc(II) phthalocyanine exhibited the highest inhibition against *S. aureus* (91.28%) and *P. aeruginosa* (85.74) at 250 mg/L. Therefore, compound TCuPc can be considered a promising candidate for the development of novel antibiofilm agents targeting resistant bacterial infections.

#### 2.2.5. Surface Antimicrobial Activity with and Without Photodynamic Therapy

**Figure 7:**
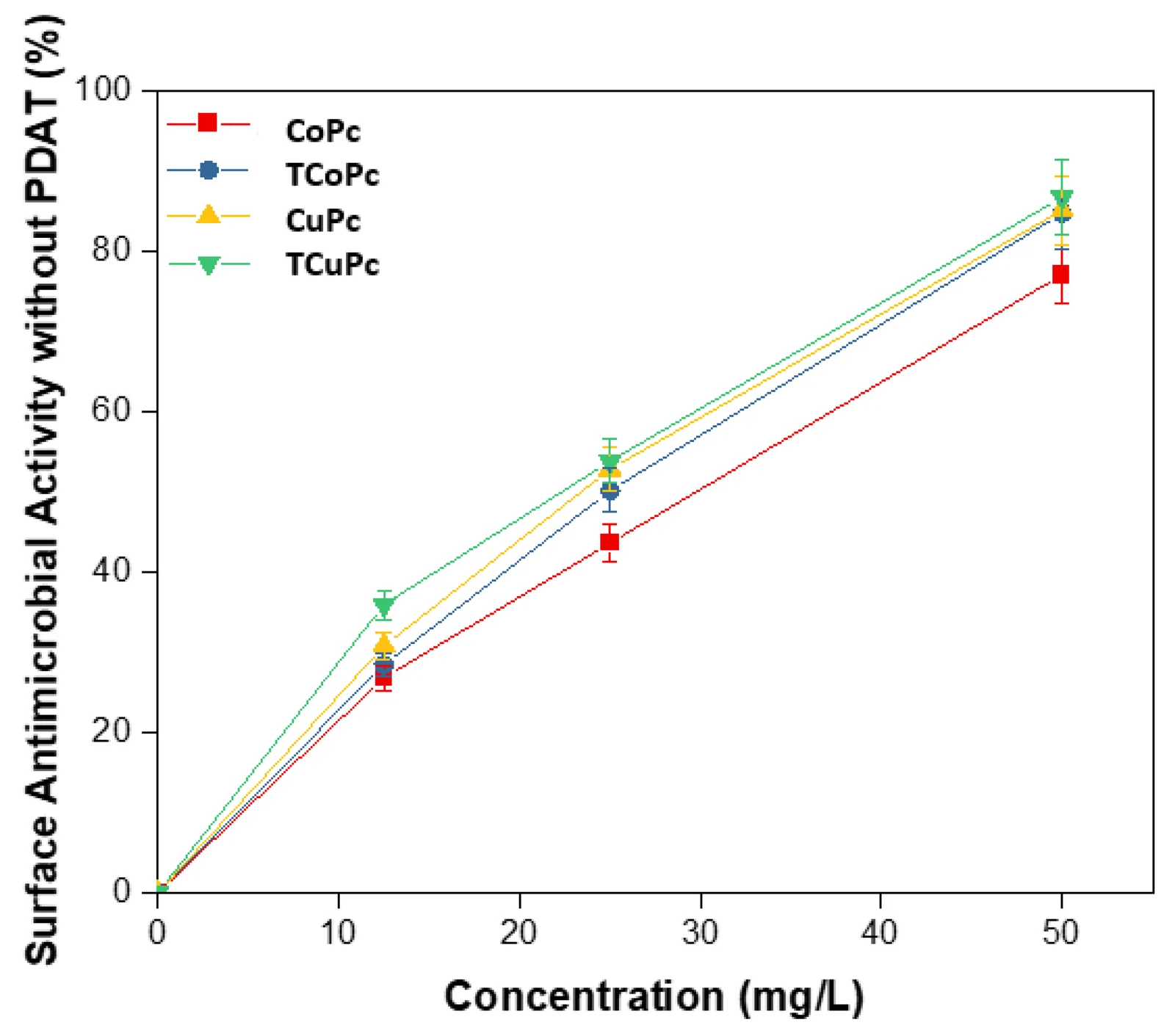
Surface antimicrobial activities of compounds (CoPc, TCoPc, CuPc, and TCuPc) without PDaT.

In this study, the surface antimicrobial activities of compounds (CoPc, TCoPc, CuPc, and TCuPc) were investigated at different concentrations (12.5–50 mg/L). The Gram-negative bacterium *E. coli* was used as a model microorganism. All the phthalocyanines exhibited increasing inhibitory activities with increasing concentration (Figure 7). Additionally, the results are given in Table 4. The inhibition activities were 26.85% ± 1.54 for CoPc, 28.44% ± 1.48 for TCoPc, 30.79% ± 1.69 for CuPc, and 35.93% ± 1.85 for TCuPc at 12.5 mg/L, respectively. Additionally, all the complexes exhibited high inhibition rates at 50 mg/L. The surface antimicrobial inhibition activities of compounds (CoPc, TCoPc, CuPc, and TCuPc) were determined to be 77.12% ± 3.67, 84.79% ± 4.56, 85.14% ± 4.35, and 86.78% ± 4.62, respectively.

**Figure 8:**
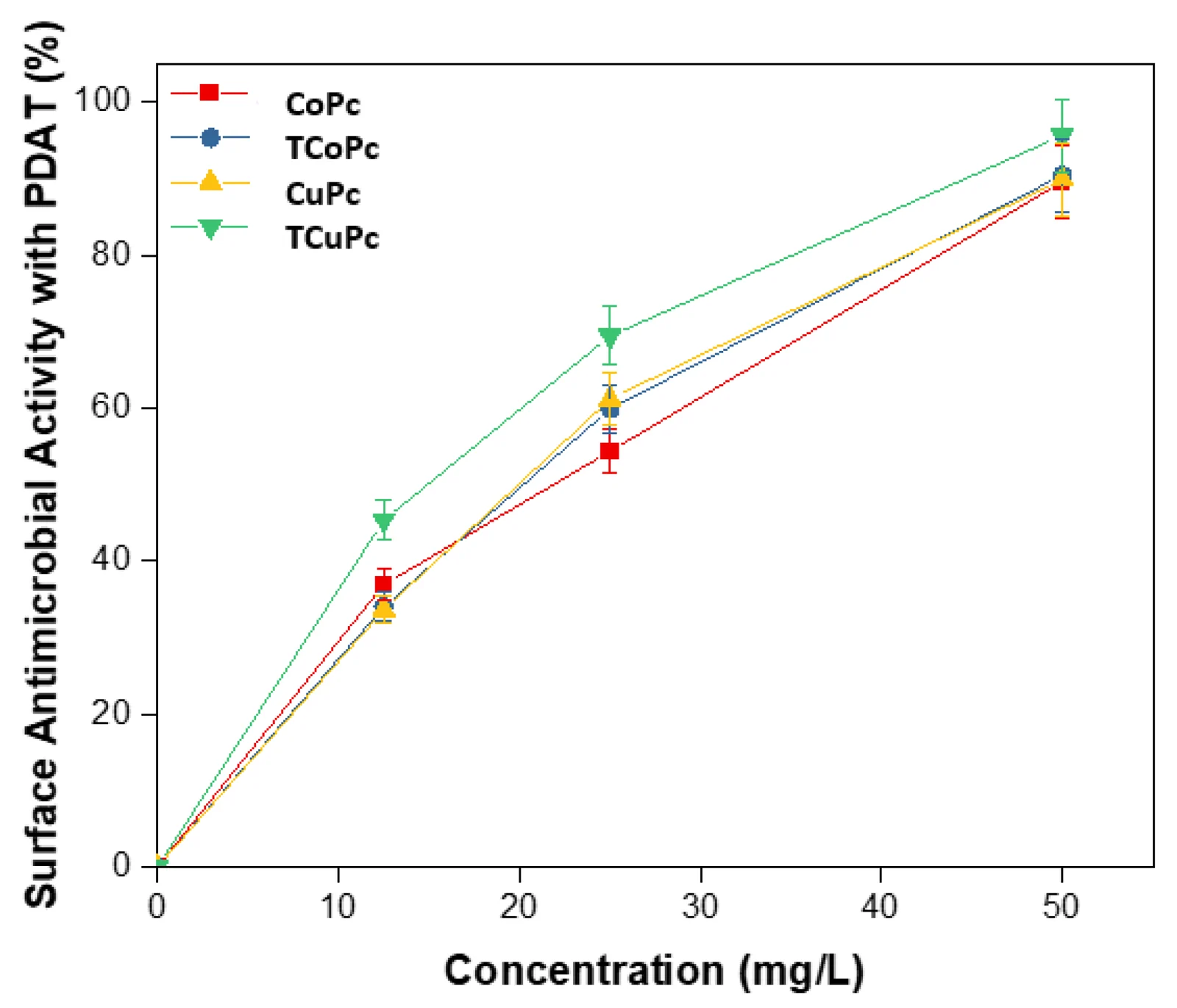
Surface antimicrobial activities of compounds (CoPc, TCoPc, CuPc, and TCuPc) with PDaT.

The surface antimicrobial activities of compounds (CoPc, TCoPc, CuPc, and TCuPc) against surface microbial viability were also evaluated under photodynamic conditions (Figure 8). The results are listed in Table 4. Accordingly, the inhibition values increased after light activation. The photodynamic inhibition values for compounds (CoPc, TCoPc, CuPc, and TCuPc) were 36.94% ± 1.91 and 54.37% ± 2.87 for CoPc, 34.06% ± 1.79 and 59.92% ± 3.18 for TCoPc, 33.62% ± 1.76 and 61.21% ± 3.34 for CuPc, and 45.38% ± 2.69 and 69.47% ± 3.81 for TCuPc at concentrations of 12.5 mg/L and 25 mg/L, respectively. These values are higher than those measured in the dark, confirming their photodynamic activities.

The highest inhibition was observed at 50 mg/L. The photodynamic inhibition values were 89.68% ± 4.74 for CoPc, 90.53% ± 4.82 for TCoPc, 89.97% ± 4.73 for CuPc, and 95.71% ± 4.76 for TCuPc. Therefore, the ranking of photodynamic activity was TCuPc > TCoPc ≈ CuPc > CoPc. Vallejo et al. (2021) noted that immobilizing zinc(II) phthalocyanines on semiconductor surfaces can be an important approach in this field [63]. They synthesized thin films of titanium dioxide with zinc(II)-tetracarboxyphthalocyanine, which exhibited strong antimicrobial activity against methicillin-resistant *S. aureus* under visible light. They also explained that singlet oxygen and other ROS generated by photoexcitation damaged the bacterial cell membrane and intracellular components in this system, leading to microbial inactivation. Ballatore et al. (2025) focused on developing self-sterilizing photodynamic surfaces by stably immobilizing zinc(II) porphyrin derivatives [64]. They aimed to prevent bacterial colonization by ensuring continuous ROS production via light activation of these surfaces. They reported that photodynamic surfaces were particularly effective at preventing biofilm formation and reduced bacterial populations by 99.7% and 99.99% with ZnTCP-C60 and ZnACP, respectively. Bayat and Karimi (2019) tested the photodynamic activity of zinc phthalocyanine conjugates immobilized in a chitosan hydrogel matrix against Gram-negative bacteria [65]. They stated that such hybrid systems had potential applications in biomedical surface coatings owing to their biocompatibility and high singlet-oxygen production. Compared with the literature, the findings demonstrated that the newly synthesized metal phthalocyanines can effectively reduce the viability of microorganisms on surfaces under photodynamic conditions and exhibit photosensitizing potential.

## 3. Materials and Methods

All materials used in this study were of high purity. Chemicals, including 4-(4-Fluorophenyl)thiazole-2-thiol (≥99.9%), copper(II) chloride (99%), cobalt(II) chloride (≥98%), and potassium carbonate (99%), were purchased from Sigma-Aldrich, Schnelldorf, Germany. Anhydrous N,N-dimethylformamide (99.8%), N,N-dimethylaminoethanol (≥99.5%), ethanol (≥99.8%), tetrahydrofuran (≥99.9%), and *n*-hexane (99%) were obtained from the same company. Compounds CoPc and CuPc were prepared as described in the literature, with some modifications [14,15,16].

Safety and waste management: N,N-dimethylformamide and N,N-dimethylaminoethanol were used as solvents and required proper precautions. N,N-dimethylformamide is a suspected reproductive toxicant that can be absorbed through the skin; it was handled in a certified fume hood with nitrile gloves, safety goggles, and a lab coat. Contact with strong oxidizers and hydrolysis-sensitive reagents was avoided. N,N-dimethylaminoethanol is corrosive and irritating to the skin, eyes, and respiratory tract; it was handled in a fume hood with appropriate PPE and kept away from strong acids and oxidizing agents. All solvent waste, including used N,N-dimethylformamide, N,N-dimethylaminoethanol, and contaminated materials, was collected separately in labeled, sealed containers as organic solvent waste and disposed of through the institution’s licensed hazardous waste service, in accordance with local regulations. In Turkey, waste was managed by İZAYDAŞ (İzmit Atık ve Artıkları Değerlendirme A.Ş.) in compliance with the Ministry of Environment, Urbanization and Climate Change’s hazardous waste regulations. No DMF or DMAE waste was discharged into regular drains.

### 3.1. Synthesis and Characterization

#### 3.1.1. Synthetic Procedure for 3-((4-(4-fluorophenyl)thiazol-2-yl)thio)phthalonitrile

3-nitro phthalonitrile (0.50 g, 2.89 mmol) and 4-(4-fluorophenyl)thiazole-2-thiol (0.700 g, 3.30 mmol) were dissolved in dry N,N-dimethylformamide (10 mL). After the addition of potassium carbonate (1.00 g, 7.25 mmol), the reaction mixture was stirred at 70 °C for 72 h under an inert atmosphere. The synthetic process was monitored using thin-layer chromatography, and loss of the precursors confirmed reaction completion. The mixture was cooled to room temperature, poured into iced water (50 mL), and stirred for 2 h. The precipitate was filtered, washed several times with water, and dried. The product was purified by recrystallization of the newly synthesized phthalonitrile from ethanol. Molecular formula: C_17_H_8_FN_3_S_2_. Color: yellow powder. Solubility: dimethylsulfoxide, N,N-dimethylformamide, tetrahydrofuran, *n*-pentanol, *n*-hexanol, N,N-dimethylaminoethanol. Melting point: >250 °C. Yield: 0.66 g (68%). ^1^H NMR (500 MHz; DMSO-d_6_): δ (ppm) 8.27 (s, 1H), 8.22–8.19 (d, 1H), 8.10–8.07 (d, 1H), 7.98–7.92 (m, 5H). FT-IR υ (cm^−1^): 3043 (aromatic C–H), 2227 (nitrile C≡N), 1251 (C-F), 1046 (C-S-C). Anal. calcd. for C_17_H_8_FN_3_S_2_: C 60.52, H 2.39, N 12.45%, found: C 60.59, H 2.40, N 12.42%.

#### 3.1.2. General Synthetic Procedure for Metal Phthalocyanines (TCoPc and TCuPc)

3-((4-(4-fluorophenyl)thiazol-2-yl)thio)phthalonitrile (0.100 g, 0.296 mmol) and the related dry metal chlorides {CoCl_2_ (0.011 g, 0.088 mmol) and CuCl_2_ (0.012 g, 0.089 mmol)} were stirred in N,N-dimethyl aminoethanol (3 mL) at reflux under an inert atmosphere for 24 h. The reaction progress was monitored by performing thin-layer chromatography, and loss of the phthalonitrile indicated reaction completion. The reaction mixture was cooled to room temperature, treated with iced water (10 mL), and stirred for 1 h. The precipitate was filtered, washed several times with hot *n*-hexane, and dried. The product was purified by column chromatography on silica gel as the stationary phase and a mixture of *n*-hexane: tetrahydrofuran (3:1) as the eluent.

TCoPc: Molecular formula: C_68_H_32_CoF_4_N_12_S_8_. Color: bluish-green powder. Solubility: dimethylsulfoxide, N,N-dimethylformamide, tetrahydrofuran, dichloromethane, chloroform, ethyl acetate. Melting point: >250 °C. Yield: 0.041 g (39%). FT-IR υ (cm^−1^): 3031 (C–H aromatic), 1249 (C-F), 1070 (C-S-C). UV–vis (DMSO): λ_max_ nm (log ε) 357 (4.54), 681 (4.97). MS *m*/*z* calcd. 1408.51 [M]^+^ found 1327.90 [M − C_9_H_5_FNS_2_ + 2K + 3OH]^+^.

TCuPc: Molecular formula: C_68_H_32_CuF_4_N_12_S_8_. Color: green powder. Solubility: dimethylsulfoxide, N,N-dimethylformamide, tetrahydrofuran, dichloromethane, chloroform, ethyl acetate. Melting point: >250 °C. Yield: 0.045 g (43%). FT-IR υ (cm^−1^): 3038 (C–H aromatic), 1251 (C-F), 1075 (C-S-C). UV–vis (DMSO): λ_max_ nm (log ε) 351 (4.79), 684 (4.95). MS *m*/*z* calcd. 1413.12 [M]^+^ found 1518.61 [M + 3Na + 2H_2_O]^+^.

### 3.2. Biological Studies

All biological activities were tested in triplicate, and standard deviations were calculated.

#### 3.2.1. DNA Cleavage Activity

The DNA cleavage activity of compounds (CoPc, TCoPc, CuPc, and TCuPc) was evaluated by applying agarose gel electrophoresis. pBR322 plasmid DNA was used as the target molecule. 15 µL of each compound (CoPc, TCoPc, CuPc, or TCuPc) was combined with 5 µL of pBR322 plasmid DNA (0.1 μg/μL) in PCR tubes and incubated at 37 °C for 2 h in the dark. Then, a 1% agarose gel was prepared and placed in an electrophoresis chamber filled with TAE buffer. Each reaction mixture was blended with loading dye and loaded onto a 1% (*w*/*v*) agarose gel pre-stained with ethidium bromide (0.5 µg mL^−1^). Untreated plasmid DNA with DMSO, incubated under identical conditions without any test compound, served as the negative control. Electrophoresis was carried out for 1 h at 100 V to separate DNA molecules. After completion of electrophoresis, the gel was removed, and DNA bands were visualized using a UV transilluminator (UVP^®^ Model LMS-20 3UV™ Benchtop UV Transilluminator, Upland, CA, USA).

#### 3.2.2. Antioxidant Activity

To determine the antioxidant activity of compounds (CoPc, TCoPc, CuPc, and TCuPc), the DPPH radical-scavenging assay was performed as described in the literature [56]. The compounds were prepared at concentrations ranging from 6.25 mg/L to 100 mg/L by serial dilution of the stock solution. 0.5 mL of each Pc compound solution and 2 mL of DPPH solution were placed in separate test tubes. The solutions were mixed and shaken vigorously, then incubated at 25 °C in the dark for 30 min. The absorbance of each sample was measured at 517 nm using a UV-vis spectrophotometer. Ascorbic acid and Trolox were used as controls. DPPH inhibition values were calculated as percentages using the following Formula (1).

DPPH Inhibition (%) = [(A(control) − A(sample)/A(control)] × 100(1)

#### 3.2.3. Antimicrobial Activity

The minimum inhibitory concentration (MIC) is the lowest concentration of a compound required to completely inhibit visible microbial growth. The antimicrobial potential of compounds (CoPc, TCoPc, CuPc, and TCuPc was assessed using a broth microdilution assay. Test microorganisms included *Escherichia coli* (ATCC 10536), *Pseudomonas aeruginosa* (ATCC 9027), *Legionella pneumophila* subsp. *Pneumophila* (ATCC 33152), *Bacillus cereus* (ATCC 11778), *Enterococcus hirae* (ATCC 10541), *Staphylococcus aureus* (ATCC 6538), *Candida albicans* (ATCC 10231), and *Candida tropicalis* (ATCC 750). The day before testing, the microorganisms were cultured in Nutrient Broth (NB) at 37 °C for 18 h. First, 150 µL of NB was added to the plates, and then serial 1:1 dilutions of the compounds were prepared in 96-well microplates. After dilution, 2.8 × 10^8^ CFU/mL of each microorganism was added, and the plates were incubated at 37 °C for 24 h. MIC values were then determined. Ampicillin was used as a positive control for bacteria, and fluconazole was used as a positive control for microfungi. DMSO was also used as a negative control.

#### 3.2.4. Biofilm Inhibitory Activity

The inhibitory effects of compounds (CoPc, TCoPc, CuPc, and TCuPc) on biofilm formation were evaluated against *S. aureus* and *P. aeruginosa*. Experiments were conducted in 24-well plates containing different concentrations of Nutrient Broth (NB) (50, 100, and 200 mg/L). After inoculation with freshly prepared bacterial suspensions, the plates were incubated at 37.5 °C for 72 h to promote bacterial adhesion and biofilm formation. Planktonic cells were removed, and the wells were gently cleaned 2 times with 500 µL of phosphate-buffered saline (PBS). The plates were then air-dried for approximately 30 min. The remaining biofilms were stained with 200 µL of 1% crystal violet solution and incubated for 1 h. The excess dye was removed, and the wells were washed with PBS to remove any residual dye. The bound crystal violet was then dissolved in ethanol. The plates were kept at room temperature for 15 min, after which the absorbance of the released dye was measured at 595 nm using a microplate reader (BioTek ELx800, Winooski, VT, USA). The biofilm inhibition percentages were calculated according to Equation (2).

Biofilm Inhibition (%) = [(A(control) − A(sample)/A(control)] × 100(2)

#### 3.2.5. Surface Antimicrobial Activity with and Without Photodynamic Therapy

The antibacterial activities of the prepared surfaces were determined using a surface-contact method, widely used in the literature with some modifications [66,67]. The Gram-negative bacterium *E. coli* was used as a model microorganism to evaluate antibacterial activity. Bacterial cultures were grown by incubation in Nutrient Broth at 37 °C for 18 h. After incubation, the bacterial suspensions were diluted with sterile physiological saline to approximately 1 × 10^8^ CFU/mL. Before the experiment, the surfaces were sterilized under ultraviolet light for 30 min to prevent surface contamination. Different concentrations of each compound were prepared. Then, 100 µL of each compound solution was applied to each surface and spread evenly, followed by 100 µL of the prepared bacterial suspension, which was spread evenly using a sterile loop. The inoculated surfaces were incubated in sterile Petri dishes at 37 °C for 24 h. The samples were then placed in an oven with 10 mL of phosphate-buffered saline (PBS) and shaken at 120 rpm for 30 min to recover surface-associated bacteria. Serial ten-fold dilutions were prepared from the resulting bacterial suspension, and 100 µL of each dilution was plated onto Nutrient Agar plates using the spread method. For the photodynamic therapy (PDT) study, the surfaces were first irradiated with LED light for 30 min before bacterial exposure, and the same procedure was then repeated. A red-orange light-emitting diode was used at 632 ± 2 nm with a dosage of 12 J cm^−2^. Colonies were counted after incubating the plates at 37 °C for 24 h. The results were expressed as colony-forming units (CFU/mL). Antibacterial activity was evaluated by comparing the number of bacteria on control surfaces (inoculated only with bacteria) to that on test surfaces (inoculated with bacteria and test compounds). Antibacterial % efficacy was calculated using the following Equation (3):

Surface Antimicrobial Activity (%) = [(A(control) − A(sample)/A(control)] × 100(3)

A(control) is the average number of live bacteria recovered from the control surfaces after incubation. A(sample) is the average number of live bacteria recovered from the antibacterial surfaces.

## 4. Conclusions

In this study, a new thiazole-containing phthalonitrile derivative and its metal phthalocyanines, {cobalt(II) and copper(II)}, were successfully prepared and structurally characterized. To examine the effects of the metal ion and substituent on the biological characteristics of the phthalocyanine ring, unsubstituted cobalt and copper phthalocyanines were also prepared using established methods. The tested derivatives (CoPc, TCoPc, CuPc, and TCuPc) exhibited significant DNA-cleavage, antioxidant, antimicrobial, antibiofilm, and photodynamic activities. They all caused complete DNA degradation at 125–250 mg/L. Antioxidant activity was consistent across doses, ranging from 16.44 to 69.54% at 6.25 mg/L to 28.70–77.91% at 100 mg/L, with the order TCuPc > TCoPc > CoPc > CuPc. TCuPc demonstrated the strongest antimicrobial effects, with MIC values as low as 16 mg/L against *S. aureus*, *L. pneumophila*, *and B. cereus*, and 32 mg/L against *E. hirae*, *P. aeruginosa*, *and E. coli*. CoPc and TCoPc showed moderate activity (MIC 64–128 mg/L). Additionally, TCuPc achieved the highest biofilm inhibition, up to 92.35% against *S. aureus* and 87.24% against *P. aeruginosa* at 200 mg/L. Its surface antimicrobial inhibition reached 86.78% at 50 mg/L in the dark and increased to 95.71% under photodynamic (light-activated) conditions, underscoring its superior photodynamic efficiency. Overall, these findings suggest that TCuPc is the most promising candidate for antimicrobial surface applications and photodynamic therapy.

## Figures and Tables

**Table 1:** Antioxidant activities of compounds (CoPc, TCoPc, CuPc, and TCuPc).

Concentration (mg/L)	DPPH Radical Scavenging Activity (%)				
	6.25	12.5	25	50	100
CoPc	16.44 ± 0.89	21.25 ± 1.27	25.77 ± 1.43	26.07± 1.57	28.70 ± 1.48
TCoPc	66.66 ± 3.49	66.37 ± 3.56	67.70 ± 3.44	68.88 ± 3.38	70.07 ± 3.76
CuPc	8.00 ± 0.45	8.88 ± 0.41	9.62 ± 0.49	9.92 ± 0.52	10.37 ± 0.58
TCuPc	69.54 ± 3.67	70.55 ± 3.43	72.18 ± 3.61	74.92 ± 3.52	77.91 ± 3.68
Ascorbic acid	80.73 ± 5.19	87.57 ± 5.45	91.08 ± 5.76	100 ± 6.18	100 ± 6.21
Trolox	82.11 ± 5.23	87.35 ± 5.39	92.79 ± 5.51	100 ± 5.91	100 ± 6.48

**Table 2:** Antimicrobial activities of compounds (CoPc, TCoPc, CuPc, and TCuPc).

Microorganisms	MICs (mg/L)					
	CoPc	TCoPc	CuPc	TCuPc	FCZ	AMP
*E. coli*	64	64	64	32	-	0.5
*P. aeruginosa*	128	128	64	32	-	0.5
*L. pneumophila*	32	32	32	16	-	0.5
*E. hirae*	128	128	64	32	-	0.5
*B. cereus*	256	128	128	16	-	1
*S. aureus*	128	128	64	16	-	0.5
*C. albicans*	64	64	64	64	1	-
*C. tropicalis*	128	128	64	64	1	-

FCZ: Fluconazole; AMP: Ampicillin.

**Table 3:** Biofilm inhibitory activities of compounds (CoPc, TCoPc, CuPc, and TCuPc) against biofilm formation of *S. aureus* and *P. aeruginosa.*

Concentartion (mg/L)	Against *S. aureus* (%)			Against *P. aeruginosa* (%)		
	50	100	200	50	100	200
CoPc	22.58 ± 1.27	32.71 ± 1.45	56.15 ± 2.84	25.93 ± 1.39	43.79 ± 2.46	70.28 ± 3.68
TCoPc	26.83 ± 1.51	47.64 ± 2.63	68.91 ± 3.71	29.11 ± 1.59	50.74 ± 2.79	73.68 ± 3.65
CuPc	29.67 ± 1.67	54.26 ± 2.79	77.42 ± 3.88	31.37 ± 1.67	58.04 ± 2.96	76.42 ± 3.72
TCuPc	33.48 ± 1.74	63.27 ± 3.41	92.35 ± 4.86	33.15 ± 1.75	60.53 ± 3.21	87.24 ± 4.67

**Table 4:** Surface antimicrobial activities of compounds (CoPc, TCoPc, CuPc, and TCuPc) against *E. coli*.

Concentartion (mg/L)	Without PDAT (%)			With PDAT (%)		
	12.5	25	50	12.5	25	50
CoPc	26.85 ± 1.54	43.64 ± 2.39	77.12 ± 3.67	36.94 ± 1.91	54.37 ± 2.87	89.68 ± 4.74
TCoPc	28.44 ± 1.48	50.18 ± 2.75	84.79 ± 4.56	34.06 ± 1.79	59.92 ± 3.18	90.53 ± 4.82
CuPc	30.79 ± 1.69	52.86 ± 2.81	85.14 ± 4.35	33.62 ± 1.76	61.21 ± 3.34	89.97 ± 4.73
TCuPc	35.93 ± 1.85	53.84 ± 2.76	86.78 ± 4.62	45.38 ± 2.69	69.47 ± 3.81	95.71 ± 4.76

## Data Availability

Data is contained within the article.

## Supplementary Materials

The following supporting information can be downloaded at: https://www.mdpi.com/article/10.3390/ph19091459/s1, Figure S1: FT-IR spectra of 3-((4-(4-fluorophenyl)thiazol-2-yl)thio)phthalonitrile (a), TCoPc (b), and TCuPc (c); Figure S2: ^1^H NMR spectrum of 3-((4-(4-fluorophenyl)thiazol-2-yl)thio)phthalonitrile; Figure S3: UV-vis spectrum of compound TCoPc (Concentration: 10 micromolar, DMSO); Figure S4: UV-vis spectrum of compound TCuPc (Concentration: 10 micromolar, DMSO); Figure S5: MALDI-TOF spectrum of compound TCoPc; Figure S6: MALDI-TOF spectrum of compound TCuPc.

## Abbreviations

The following abbreviations are used in this manuscript:

Pcs	Phthalocyanines
ROS	Reactive oxygen species
MIC	Minimum inhibitory concentration
PDAT	Photodynamic antibacterial therapy
