# Supplementary material for: In Vitro Biological Properties of Thiazole-Containing Cobalt(II) and Copper(II) Phthalocyanines: DNA Cleavage, Antioxidant, Antimicrobial, Antibiofilm, and Surface Anti-Escherichia coli Activities
Source: Pharmaceuticals (Basel). 2026 Sep 14;19(9):1459. doi: 10.3390/ph19091459 (PMC13610550; doi:10.3390/ph19091459)
Supplement: Supplementary file 1 [file pharmaceuticals-19-01459-s001.zip › pharmaceuticals-4543242-supplementary.pdf]

Supplementary Materials

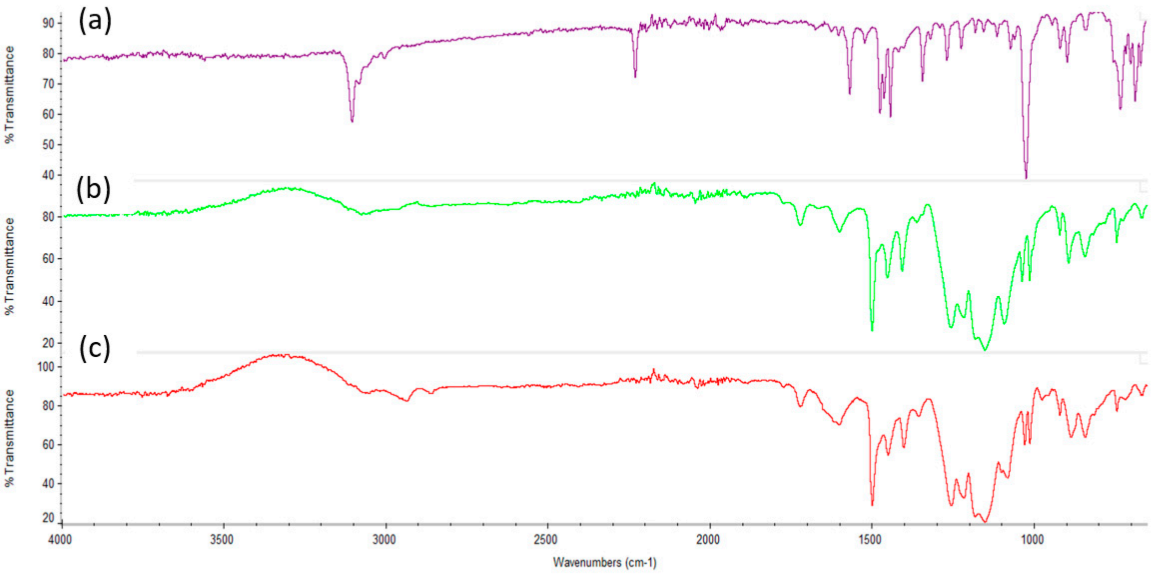

Figure S1. FT-IR spectra of 3-((4-(4-fluorophenyl)thiazol-2-yl)thio)phthalonitrile (**a**), TCoPc (**b**), and TCuPc (**c**).

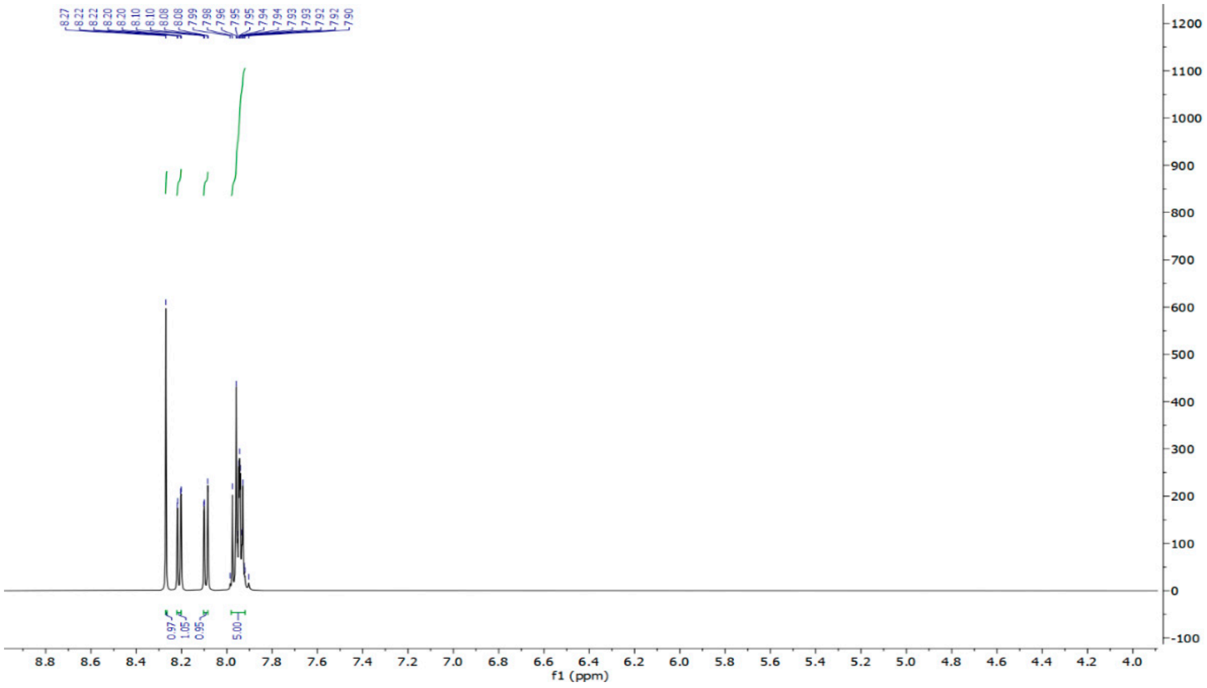

Figure S2. ^{1}H NMR spectrum of 3-((4-(4-fluorophenyl)thiazol-2-yl)thio)phthalonitrile.

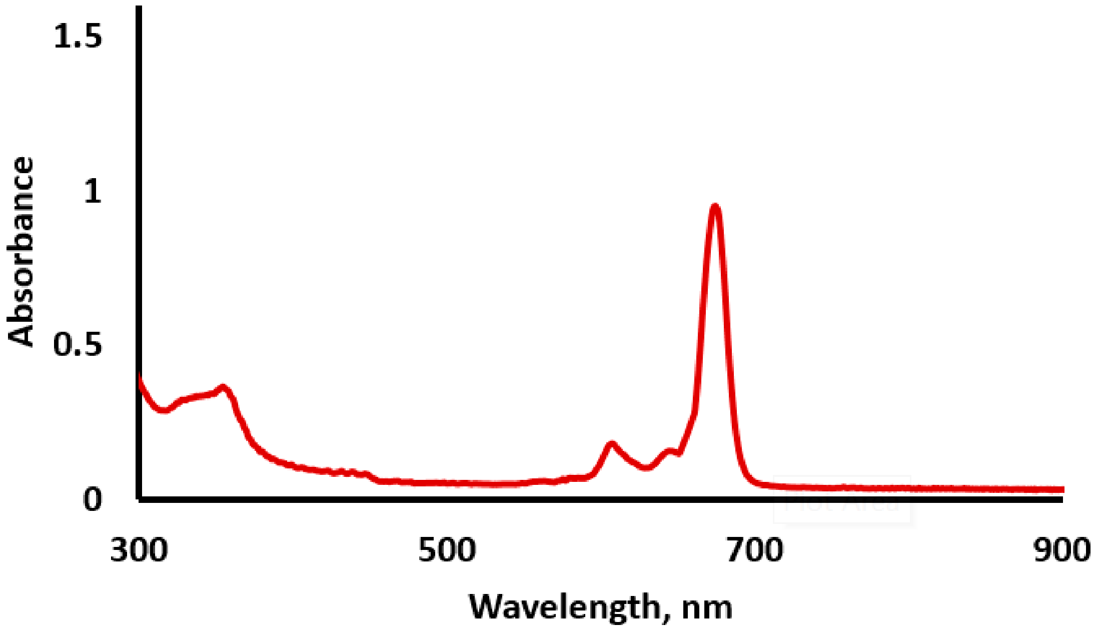

Figure S3. UV-vis spectrum of compound TCoPc (Concentration: 10 micromolar, DMSO).

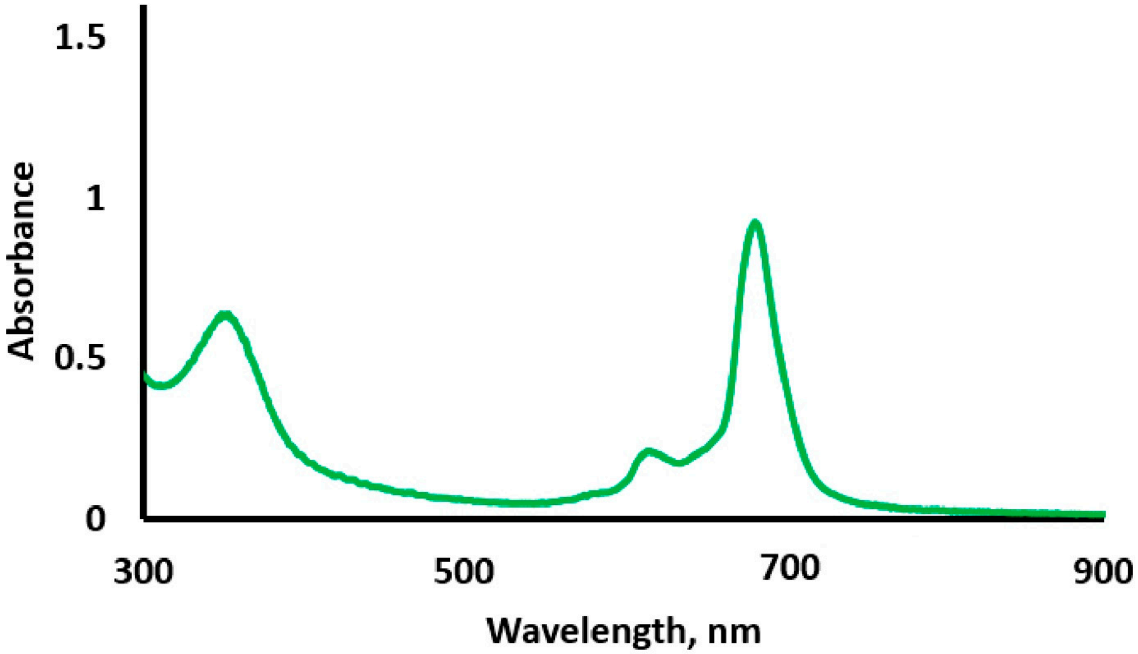

Figure S4. UV-vis spectrum of compound TCuPc (Concentration: 10 micromolar, DMSO).

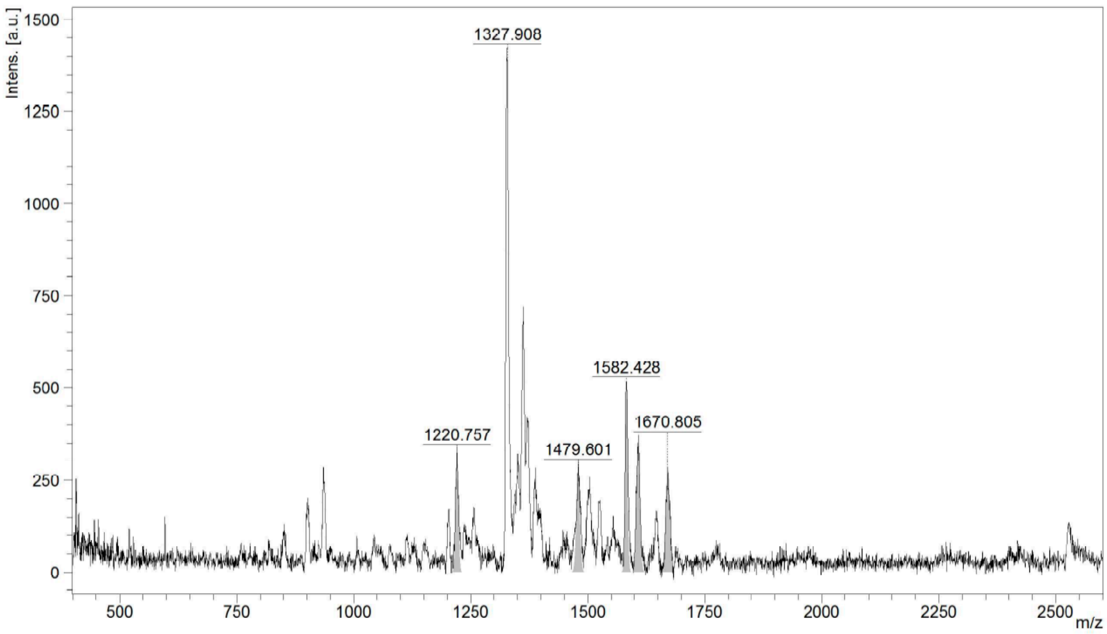

Figure S5. MALDI-TOF spectrum of compound TCoPc.

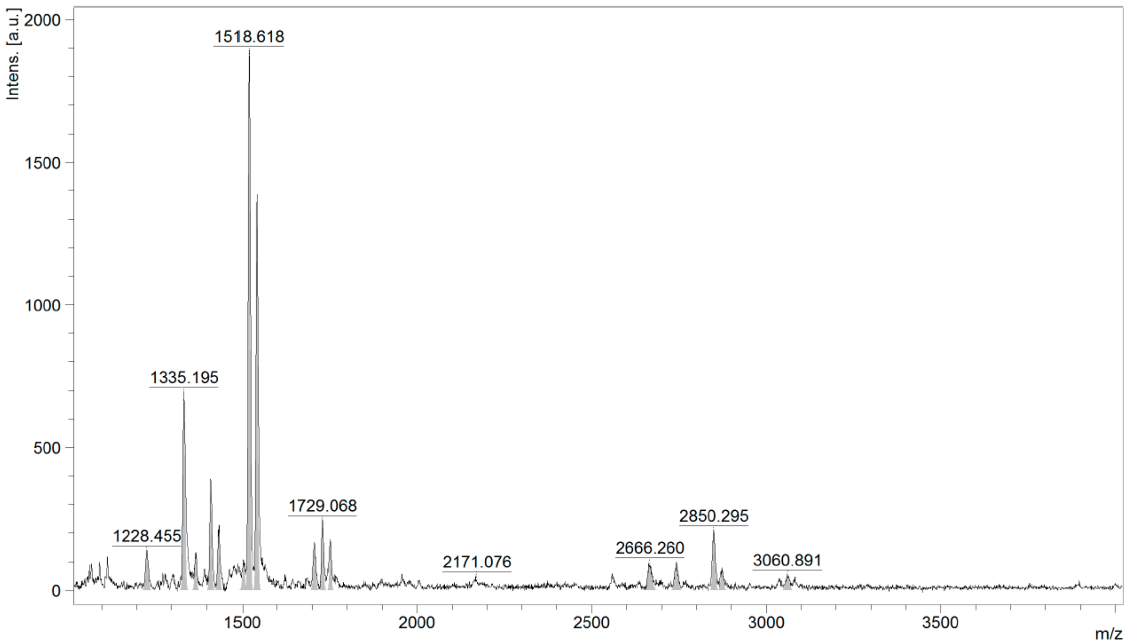

Figure S6. MALDI-TOF spectrum of compound TCuPc.
